# Letter to the Editor on “Chrysotile and rock wool fibers induce chromosome aberrations and DNA damage in V79 lung fibroblast cells”

**DOI:** 10.1007/s11356-018-1210-4

**Published:** 2018-01-18

**Authors:** John G. Hadley, Angus E. Crane

**Affiliations:** North America Insulation Manufacturers Association, 11 Canal Center Plaza, #103, Alexandria, VA 22314 USA

*Environmental Science and Pollution Research*’s recently published article, “Chrysotile and rock wool fibers induce chromosome aberrations and DNA damage in V79 lung fibroblast cells,” has come to the attention of the North American Insulation Manufacturers Association (NAIMA). NAIMA is the association for North American manufacturers of fiber glass, rock wool, and slag wool insulation products.

The above-referenced article claims that rock wool insulation fibers cause DNA damage. The article is based on a study that was conducted with chrysotile and “an artificial substitute, rock wool fiber were prepared as suspensions and were tested at concentrations of 50, 100, and 200 μg/ml in V79 lung fibroblasts.” (Cui et al. 2017) The test results found: “Chromosome aberrations were detected by micronucleus assay after exposure for 24 h, and DNA damage were estimated by single cell gel electrophoresis after exposure for 12, 24, or 48 h.”^1^ Moreover, the fiber preparation, as described in the article, raises serious concerns: “Fibers were crushed in a ceramic mortar, then cut and ground to 2-3 mm. This material was suspended in ethyl alcohol and ground for 12 h in a horizontal ball mill, and then passed through a 400-mesh screen.”^2^ There is no verification that when this process was completed there were actually any fibers left; it is not inconceivable that what had been created by this rigorous process was nothing but dust, and fibers were nowhere in evidence.

As described in the above excerpt, the in vitro tests were completed in short periods of time. Because of the brevity of these tests, there is insufficient time for the testing process to differentiate between a durable fiber and a soluble fiber. It is imperative that such distinctions can be made because the asbestos fibers are durable and the rock wool fibers are soluble. The shortcomings of these in vitro tests were recognized by both the International Agency for Research on Cancer (IARC) and the United States Agency for Toxic Substances and Disease Registry (ATSDR).

Specifically, the IARC Monograph on Man-Made Vitreous Fibers, which, of course, includes rock wool fibers, cautioned about the possibility of short term tests giving misleading results:

It is important to appreciate the degree to which biopersistence plays a role in the different studies and end-points under review, as this property of fibres is thought to be critical in determining chronic toxicity and carcinogenic outcome in humans and in experimental animal systems. In-vitro assays are invariably short-term (i.e. from hours to days), and the effect of fibre durability is unlikely to be detected in such assays. [The Working Group noted that endotoxin is a potent environmental contaminant and its presence in fibre samples could enhance their ability to cause acute inflammation. The presence of endotoxin or the steps taken to inactivate it, were not always reported.] Therefore, short-term tests could give a misleading impression of possible long-term biological effects. This will most likely become manifest as a false-positive result in an in-vitro assay for long, non-biopersistent fibres. For a non-biopersistent fibre, the effects seen *in vitro* may apply only to the time interval *in vivo* before the fibre begins to undergo dissolution or breakage. In contrast, a durable fibre may show the effects much more slowly and is more likely to give rise to pathological change. (International Agency for Research on Cancer 2002)

Addressing the reliance on short-term in vitro testing to determine genetic effects, IARC again warned against such studies because of their inability to account for fiber durability:

Several caveats can be raised about these in-vitro studies: (i) these assays are short term and do not address issues related to fibre dissolution or biopersistence; and (ii) relatively high levels of man-made vitreous fibres on a mass basis have been studied, and the relevance to in-vivo exposure levels is questionable.^3^

IARC further finds that there are no studies to correlate genotoxic end-points with man-made vitreous fibers:

The occurrence of mutations and some forms of DNA/chromosomal damage may be related to the production of activated oxygen species which have been detected in cell-free systems and in cells exposed to man-made vitreous fibres. Chromosomal and nuclear abnormalities may also be related to the impairment of cell division by the fibres. While reactive oxygen species are produced by either non-fibrous or fibrous particles, cell cycle-associated chromosomal and nuclear abnormalities appear to be a specific response to exposure to fibres. Despite the fact that in-vitro assessment of genetic effects does not address issues related to fibre dissolution or biopersistence, these assays can determine whether a fibre has the potential to be directly genotoxic. A major gap in the current database is the absence of any studies that correlate genotoxic end-points with the pathogenic effects of man-made vitreous fibres in the same experimental animal system.^4^

The ATSDR reached the same conclusion in its Toxicological Profile on Synthetic Vitreous Fibers, which includes rock wool fibers:

No evidence for genotoxic activity of several synthetic vitreous fibers was found in bacterial mutation assays (Chamberlain and Tarmy 1977) or sister chromatid exchange assays in cultured human cells (Casey 1983). However, several cytogenetic effects have been observed in other in vitro assays. Notably absent are data on genotoxic end points following in vivo exposure of animal or humans to synthetic vitreous fibers. Results from short-term in vitro genotoxicity assays are of limited applicability to in vivo exposure scenarios because of evidence that long-term residence of synthetic vitreous fibers in the principal toxicity target, the lung, can lead to changes (dissolution, breakage into shorter fibers) that can decrease biological activities of longer fibers (IARC 2002; also see Section 3.4).^5^

It is equally important to recognize that the in vitro tests, no matter how well performed and the results meticulously recorded, are of no consequence because they do not represent a relevant route of human exposure. The gold standard for determining the hazards of respirable fibers remains the long-term animal inhalation studies at RCC^6^ and the extensive epidemiological update conducted in 2001.^7^

When such sources as IARC and ATSDR agree that genotoxicity is not an issue with synthetic vitreous fibers, it is far more authoritative than the single study under discussion. Moreover, the short-term in vitro testing conducted for this study is specifically discredited by IARC and ATSDR because the very nature of a short-term study fails to address the durability of fibers. Therefore, the conclusions reached in the article are not credible.

To further demonstrate the lack of credibility afforded in vitro studies’ ability to predict human health hazards or risks from fibers, NAIMA has quoted below from the public testimony presented before the National Toxicology Program by the eminent toxicologist Dr. Thomas W. Hesterberg. Calling upon his extensive experience and expertise, Dr. Hesterberg’s testimony provides a concise history of in vitro testing of fibers and concludes as follows:

Because all fibers compositions were toxic in the cell culture studies, it was clear that the *in vitro* cell culture models generated false positive results. It is clear that the results of the *in vitro* assays should not be considered valid for assessing human health hazards or risks from SVFs (Hesterberg and Hart 2001). Some scientists have argued that information gained from *in vitro* studies with fibers can provide useful insight into the mechanisms that cause toxicity and, indeed, tumors. Now that the importance of biopersistence to fiber pathogenicity is understood, the *in vitro* cellular approach to understanding the potential of fibers to induce cancer can be considered obsolete. Indeed, it may be that interpretation of genotoxicity effects as the key step in fiber carcinogenicity in the early studies with asbestos fibers served to deter progress in the field.

The biopersistence studies demonstrated that, in the whole animal, fiber dissolution, breakage, and lung clearance remove the non-biopersistent fiber constituents from the lung. This provided a rational explanation for why some fiber compositions do not cause lung cancer or fibrosis, even at very high exposure concentrations. As with the intra-cavity implantation studies, *in vitro* cell culture models do not include the natural filtration and clearance mechanisms found in the whole animal that has been exposed naturally by inhalation of fibers.

As Dr. Hesterberg later wrote:

In retrospect, some of the in vitro research findings were possibly over-interpreted. There is no question based on today’s knowledge that the quantities of asbestos fibers used in many of the in vitro cell studies were massive when compared to the likelihood of cells encountering one or several fibers following inhalation exposure. It is also apparent now that the design of the studies could have been improved if a substantially broader range of exposure (dose) concentrations had been studied and greater effort had been expended in linking the dose used in the in vitro studies to in vivo doses actually encountered by tissues following exposure of people or laboratory animals to airborne fibers.^8^

Based on these facts, there are serious limitations to the study on DNA damage from exposure to rock wool fibers because short-term in vitro testing does not accurately predict DNA damage since its short duration cannot account for the differences in the durability of the fibers tested.

## Footnotes

1 Cui et al (2017).

2 Cui et al (2017).

3 IARC Monograph at p. 337.

4 IARC Monograph at p. 337.

5 *Toxicological Profile for Synthetic Vitreous Fibers* (U.S. Department of Health and Human Services, Public Health Services, Agency for Toxic Substances and Disease Registry), September 2004, p. 110.

6 See, e.g., Hesterberg and Hart (2001).

7 See, e.g., Marsh et al. (2001).

8 Hesterberg et al. (2012), at 260.
